# Efficacy and safety of transarterial chemoembolization combined with lenvatinib and camrelizumab in patients with BCLC-defined stage C hepatocellular carcinoma

**DOI:** 10.3389/fonc.2023.1244341

**Published:** 2023-10-17

**Authors:** Juan Wu, Jia Zeng, Huiwen Wang, Zhuoni Huo, Xunbo Hou, Dongfeng He

**Affiliations:** Department of Interventional Radiology, Harbin Medical University Cancer Hospital, Harbin, Heilongjiang, China

**Keywords:** hepatocellular carcinoma, transarterial chemoembolization, lenvatinib, camrelizumab, efficacy

## Abstract

**Objective:**

To investigate the effectiveness and safety of combining transarterial chemoembolization (TACE) with lenvatinib and camrelizumab in patients with Barcelona Clinic Liver Cancer (BCLC) stage C hepatocellular carcinoma (HCC).

**Methods:**

We retrospectively analyzed 141 patients with BCLC stage C HCC: 57 were treated with TACE combined with lenvatinib plus camrelizumab (T + L + C), 41 were treated with TACE combined with camrelizumab (T + C), and 43 were treated with TACE (TACE). The primary outcomes were overall survival (OS) and progression-free survival (PFS), and the secondary outcomes were the objective response rate (ORR) and adverse events (AEs). Factors that affected survival were identified via Cox regression analysis.

**Results:**

Comparison of the three groups revealed a significant difference in the median overall survival (mOS), 19.8 months (95% CI 15.7–23.9) in the T + L + C combined group vs 15.7 (95% CI 13.1–18.3) months in the T + C combined group vs 9.4 (95% CI 6.2–12.5) months in the TACE group (P < 0.001). The median progression-free survival (mPFS) was significantly better in the T + L + C combination group than in the T + C combination group and the TACE group [11.4 (95% CI 7.6–15.3) months vs 8.4 (95% CI 6.2–10.5) months vs 4.8 (95% CI 3.2–6.3) months, respectively, P < 0.001)]. The objective response rate (ORR) (57.9%) and the disease control rate (DCR) (75.4%) patients in the combined T + L + C group were higher than those in the other two groups. More patients in the combined T + L + C group experienced AEs, with 16 (28.1%) patients experiencing AEs of grade 3 or higher.

**Conclusions:**

In patients with BCLC stage C HCC, those receiving the T + L + C combination demonstrated a superior survival benefit and acceptable safety profile compared patients receiving either TACE or the T + C combination.

## Introduction

According to recent statistics, primary liver cancer has become the sixth most prevalent type of cancer globally and is responsible for the third-highest number of cancer-related deaths. The most common type of primary liver cancer is hepatocellular carcinoma (HCC), which accounts for 75–85% of cases (1). Due to the insidious onset of HCC, less than 30% of patients are eligible for radical treatment at the initial diagnosis. Unfortunately, treatment options are limited for those diagnosed with advanced-stage HCC. However, transarterial chemoembolization (TACE) is a commonly used non-surgical treatment for intermediate to advanced HCC. TACE is effective in controlling tumor growth by increasing drug concentrations locally (2, 3). However, the possibility of tumor recurrence and metastasis in advanced HCC treated with TACE alone is high. TACE causes hypoxia-induced up-regulation of vascular endothelial growth factor (VEGF) and basic fibroblast growth factor (b-FGF) in tumors and maximizing tumor ischemic necrosis (4, 5). In particular, increased expression of VEGF is strongly associated with a poor prognosis of cancer (6, 7). Lenvatinib is an oral multikinase inhibitor that targets the inhibition of the expression of VEGF receptor 1-3 isoforms, FGF receptor 1-4 isoforms, platelet growth factor receptor α, RET, and KIT expression (8, 9), resulting in the inhibition of local tumor angiogenesis. In phase III studies, nivolumab and pembrolizumab showed promising clinical efficacy as first- and second-line treatments for advanced HCC, respectively, but neither met the prespecified endpoints (10, 11). Compared to limited immune checkpoint inhibitor (ICI) monotherapy for advanced HCC, ICI combined with TACE therapy delayed tumor progression and allowed downgrading of disease with access to surgical resection (12, 13). The encouraging results of the IMbrave150 trial showed that atezolizumab (anti-programmed death ligand 1) and bevacizumab (anti-VEGF) reduced the risk of disease progression by 34% and prolonged the median survival time in patients with unresectable HCC compared to sorafenib (14), and also support the synergistic antitumor effects of antiangiogenic combination immunotherapy. However, due to resistance to systemic therapy, most patients with advanced HCC do not achieve long-term survival benefits (15). Considering the synergistic anti-cancer effects of TACE combined with lenvatinib and programmed death 1 (PD-1) inhibitors, we conducted this retrospective cohort study to evaluate the efficacy and safety of T + L + C combination therapy versus T + C combination therapy and TACE alone in patients with BCLC stage C HCC.

## Materials and methods

### Patients and study design

From January 2020 to November 2021, we conducted a retrospective study of consecutive HCC patients with BCLC stage C admitted to our institution. Patients included in the study met the following criteria: age ≥18 years and ≤75 years; patients with a clear diagnosis of unresectable HCC (BCLC stage C); at least one measurable lesion as defined by the Modified Response Evaluation Criteria for Solid Tumors (mRECIST); Eastern Cooperative Oncology Group Performance Status (ECOG PS) score of 1 to 2; and adequate organ function with Child-Pugh classification of A or B. The following exclusion criteria were applied: patients who received lenvatinib for less than 4 weeks; patients who had undergone surgical resection or other local treatments such as radiotherapy, ablation, HAIC; patients with other primary malignancies or severe dysfunction of vital organs such as heart, brain, kidney and lung; patients with a history of organ transplantation and bone marrow suppression; and patients with incomplete data or failed follow-up.

### TACE procedure

A catheter was inserted through the femoral artery using the Seldinger technique, and the phrenic, common hepatic, and superior mesenteric arteries were selected for imaging to assess the blood supply to the tumor. When staining of the tumor lesions was seen, the tumor blood supply vessels were super-selected with a microcatheter, and the intrahepatic lesions were embolized using super-liquefied iodized oil, lobaplatin, and raltitrexed. Conventional TACE (cTACE) was supplemented with embolization using gelatin sponge particles or blank microspheres, whereas drug-eluting bead-based TACE (DEB-TACE) was performed using CalliSpheres-carrying microspheres loaded with injections of piroxicam hydrochloride, which were slowly injected at a rate of 1 mL/minute to embolize intrahepatic lesions until blood flow to the tumor vasculature was interrupted. Post-TACE evaluation and follow-up were performed every 4–8 weeks, and TACE was repeated as needed if residual active lesions remained in the tumor, while the hepatic function scores were maintained at a Child-Pugh classification A or B.

### Lenvatinib and camrelizumab

The day after the first TACE procedure, patients received an intravenous infusion of 200 mg camrelizumab, which was administered every 3 weeks. Lenvatinib was administered orally at 8 mg/day (weight < 60 kg) and 12 mg/day (weight ≥ 60 kg). Patients with Child-Pugh classification B received 8 mg daily, regardless of weight. In the event of lenvatinib-related toxicity, the dose was reduced for symptomatic relief [4 mg/day (weight < 60 kg) and 8 mg/day (weight ≥ 60 kg)]. According to the dosing guidelines, when adverse events (AEs) of grade ≥3 occurred, patients received a reduced dosage of the drug or discontinued therapy until symptoms resolved or were downgraded to grade 1 or 2.

### Follow-up and evaluation

The follow-up cut-off date of this study was December 31, 2022. Clinical information was retrieved through the medical record system, and all patients underwent tumor marker testing and hematologic and biochemical testing, including blood routine, coagulation index, liver and kidney function, serum ions, and thyroid function evaluation every 4–8 weeks to assess AEs. Enhanced computed tomography (CT) or magnetic resonance imaging (MRI) was performed every 2 months, and whenever residual tumors or new lesions were confirmed. Treatment was administered according to the patient’s liver function, general condition, and tumor status. The primary outcomes of this study were OS and PFS. OS was defined as the time from the first TACE procedure to death from any cause. PFS was defined as the time from the first TACE procedure to progression or death from any cause. Secondary outcomes were objective response rate (ORR) and disease control rate (DCR) as assessed according to mRECIST criteria. Safety was assessed according to version 5.0 of the National Cancer Institute Common Terminology Criteria.

### Data analysis

Data from all patients were analyzed using IBM SPSS Statistics v.25.0 software. Categorical data were expressed as frequencies, and quantitative data were expressed as mean ± standard deviation and median (interquartile spacing) of normally and skewed distributed variables, respectively. Categorical data were compared between the three groups using the χ2 test or Fisher’s exact test, as appropriate. Quantitative data were compared using a multi-sample nonparametric (Kruskal–Walls rank sum test) test. Survival curves were calculated using the Kaplan–Meier method and compared using the log-rank test. Cox risk proportional models were used for univariate and multifactorial analyses to detect independent influences on OS and PFS. P < 0.05 was considered statistically significant.

## Results

### Characteristics of the patients

**Figure 1:**
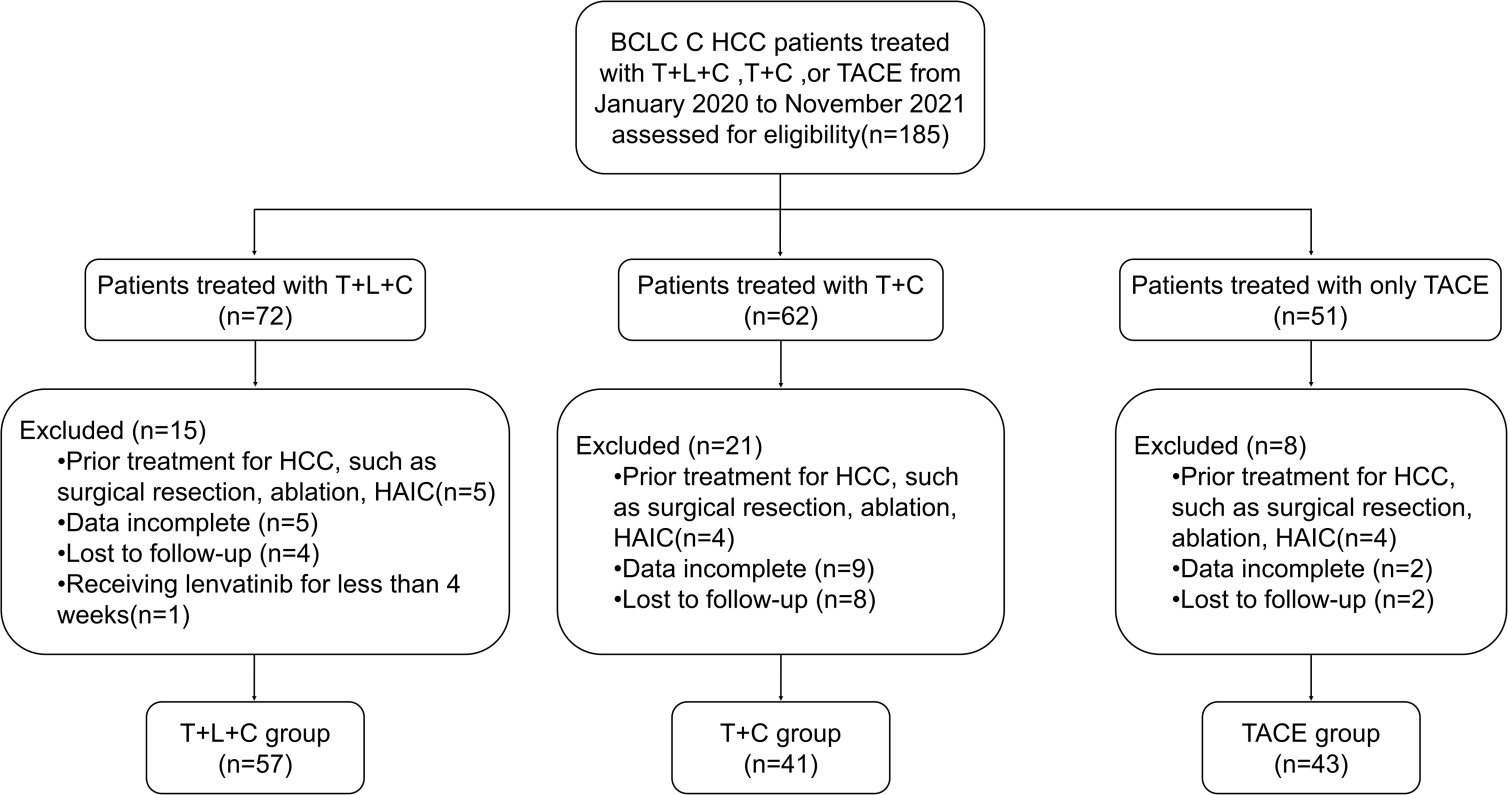
Flowchart of the patient selection process. HCC, hepatocellular carcinoma; BCLC, Barcelona Clinic Liver Cancer; T+L+C, transarterial chemoembolization combined with lenvatinib plus camrelizumab; T+C, transarterial chemoembolization combined with camrelizumab; TACE, transarterial chemoembolization.

The flow chart of this study is shown in **Figure 1**. Patients diagnosed with BCLC stage C HCC between January 2020 and November 2021 at the Affiliated Cancer Hospital of Harbin Medical University were screened for eligibility, and finally, 141 patients were included in the study (57 in the T + L + C group, 41 in the T + C group, and 43 in the TACE group). **Table 1** summarizes the baseline demographic and clinical characteristics of all patients, with the highest percentage of patients with portal vein invasion in the TACE group (69.8%) and the lowest percentage of patients with portal vein invasion in the T + C group (51.2%). Among patients with extrahepatic metastases, the highest proportion of patients in the T + L + C group had extrahepatic metastases (40.4%) and the lowest proportion in the TACE group (25.6%). There were no significant differences between the three groups in terms of demographic, clinical, or tumor characteristics. Our statistical analysis of the choice and number of TACE, the dose of iodinated oil for the first TACE, the type and dosage of chemotherapeutic drugs, and the preoperative liver function indices of the three groups showed that there were statistically significant differences between the three groups in terms of the number of TACE procedures, the dose of iodinated oil, and the dosage of lobaplatin (P < 0.05), while there were no significant differences in the preoperative liver function indexes between the three groups. Patients in the T + L + C group underwent a total of 224 TACE procedures with a median of 4 and a mean interval of 78.3 (55.5, 124.1) days between TACE sessions; patients in the T + C group underwent a total of 166 TACE procedures with a median of 4 and a mean interval of 70.2 (50.4, 99.3) days between TACE sessions; and patients in the TACE group underwent a total of 137 procedures with a median of 3 and a mean interval of 62.0 (45.0, 81.7) days between TACE sessions. There was a significant difference between the groups in the number of TACE procedures and the mean number of days between the TACE sessions (P = 0.034) (**Supplementary Table 1**).

**Table 1:** Baseline characteristics of the patients.

Characteristic	T+C (n=41)	T+L+C (n=57)	T (n=43)	P value
**Age**	55.27±10.48	53.18±9.25	53.5±9.35	0.552
**Sex**				0.326
Male	34 (82.9%)	49 (86.0%)	32 (74.4%)	
Female	7 (17.1%)	8 (14.0%)	11 (25.6%)	
**Etiology**				0.808
Hepatitis B	30 (73.2%)	44 (77.2%)	34 (79.1%)	
Others	11 (26.8%)	13 (22.8%)	9 (20.9%)	
**Cirrhosis**				0.929
Yes	30 (73.2%)	43 (75.4%)	33 (76.7%)	
No	11 (26.8%)	14 (24.6%)	10 (23.3%)	
**ECOG PS score**				0.269
1	24 (58.5%)	40 (70.2%)	32 (74.4%)	
2	17 (41.5%)	17 (29.8%)	11 (25.6%)	
**Child-Pugh class**				0.911
A	36 (87.8%)	52 (91.2%)	39 (90.7%)	
B	5 (12.2%)	5 (8.8%)	4 (9.3%)	
**ALBI Grade**				0.737
1	19 (46.3%)	25 (43.9%)	19 (44.2%)	
2	20 (48.8%)	31 (54.4%)	24 (55.8%)	
3	2(4.9%)	1 (1.8%)	0 (0.0%)	
**Number of tumors**				0.215
1	21 (51.2%)	33 (57.9%)	27 (62.8%)	
2	5 (12.2%)	4 (7.0%)	0 (0.0%)	
3	15 (36.6%)	20 (35.1%)	16 (37.2%)	
**Largest tumor size (mm)**	95 (59,110)	82 (50,111)	82 (57,98)	0.430
**portal vein invasion**				0.219
Yes	21 (51.2%)	35 (61.4%)	30 (69.8%)	
No	20 (48.8%)	22 (38.6%)	13 (30.2%)	
**Extrahepatic metastasis**				0.304
Yes	14 (34.1%)	23 (40.4%)	11 (25.6%)	
No	27 (65.9%)	34 (59.6%)	32 (74.4%)	
**AFP level (μg/L)**				0.133
≤400	24 (58.5%)	25 (43.9%)	16 (37.2%)	
>400	17 (41.5%)	32 (56.1%)	27 (62.8%)	

Data are presented as mean ± standard deviation or n (%) or median (25th-75th), T+L+C, transarterial chemoembolization combined with lenvatinib plus camrelizumab; T+C, transarterial chemoembolization combined with camrelizumab; TACE, transarterial chemoembolization; ECOG-PS, Eastern Cooperative Oncology Group performance status; ALBI, albumin-bilirubin; AFP, alpha-fetoprotein.

### Overall survival

**Figure 2:**
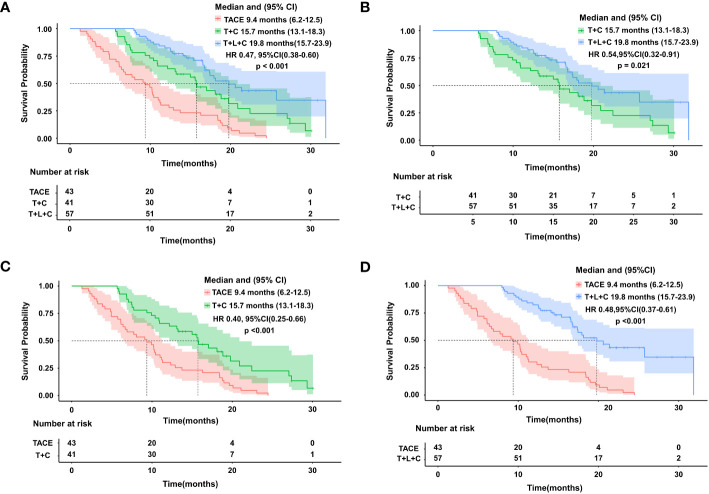
Kaplan-Meier survival curves showing OS according to treatment.Comparison of OS among the three groups **(A)**. OS comparison between T+L+C group and T+C group **(B)**. OS comparison between TACE group and T+C group **(C)**. OS comparison between T+L+C group and TACE group **(D)**.

**Figure 3:**
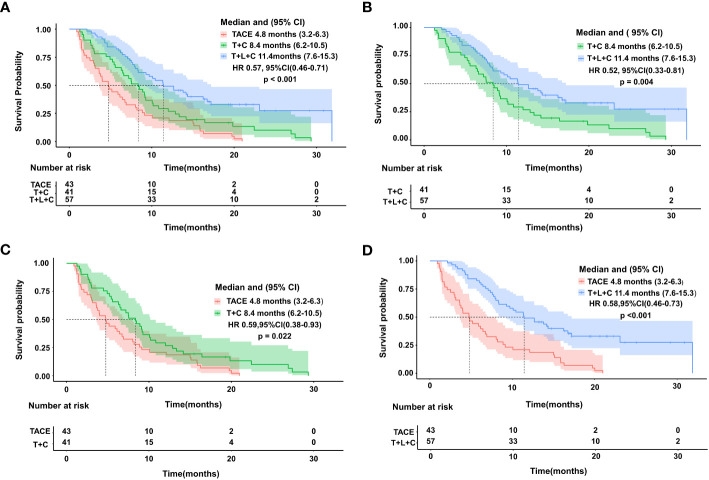
Kaplan-Meier survival curves showing PFS according to treatment.Comparison of PFS among the three groups **(A)**. PFS comparison between T+L+C group and T+C group **(B)**. PFS comparison between TACE group and T+C group **(C)**. PFS comparison between T+L+C group and TACE group **(D)**.

The entire cohort was followed for 1.3 to 31.9 months, with a median follow-up time of 14.0 months. During the follow-up period, 29 cases (50.9%) in the T + L + C group, 30 cases (73.2%) in the T + C group, and 43 cases (100.0%) in the TACE group had a mortality outcome. The median OS (mOS) was 19.8 months (95% CI 15.7–23.9 months) in the T + L + C group, 15.7 months (95% CI 13.1–18.3 months) in the T + C group, and 9.4 months (95% CI 13.1–18.3 months) in the TACE group (P < 0.001; **Figure 2**). The median PFS (mPFS) was 12.7 months (95% CI 7.6–17.8 months) in the T + L + C group, 9.1 months (95% CI 5.3–12.9 months) in the T + C group, and 4.8 months (95% CI 5.3–12.9 months) with TACE alone (P < 0.001 **Figure 3**).

### Analysis of prognostic factors

**Figure 4:**
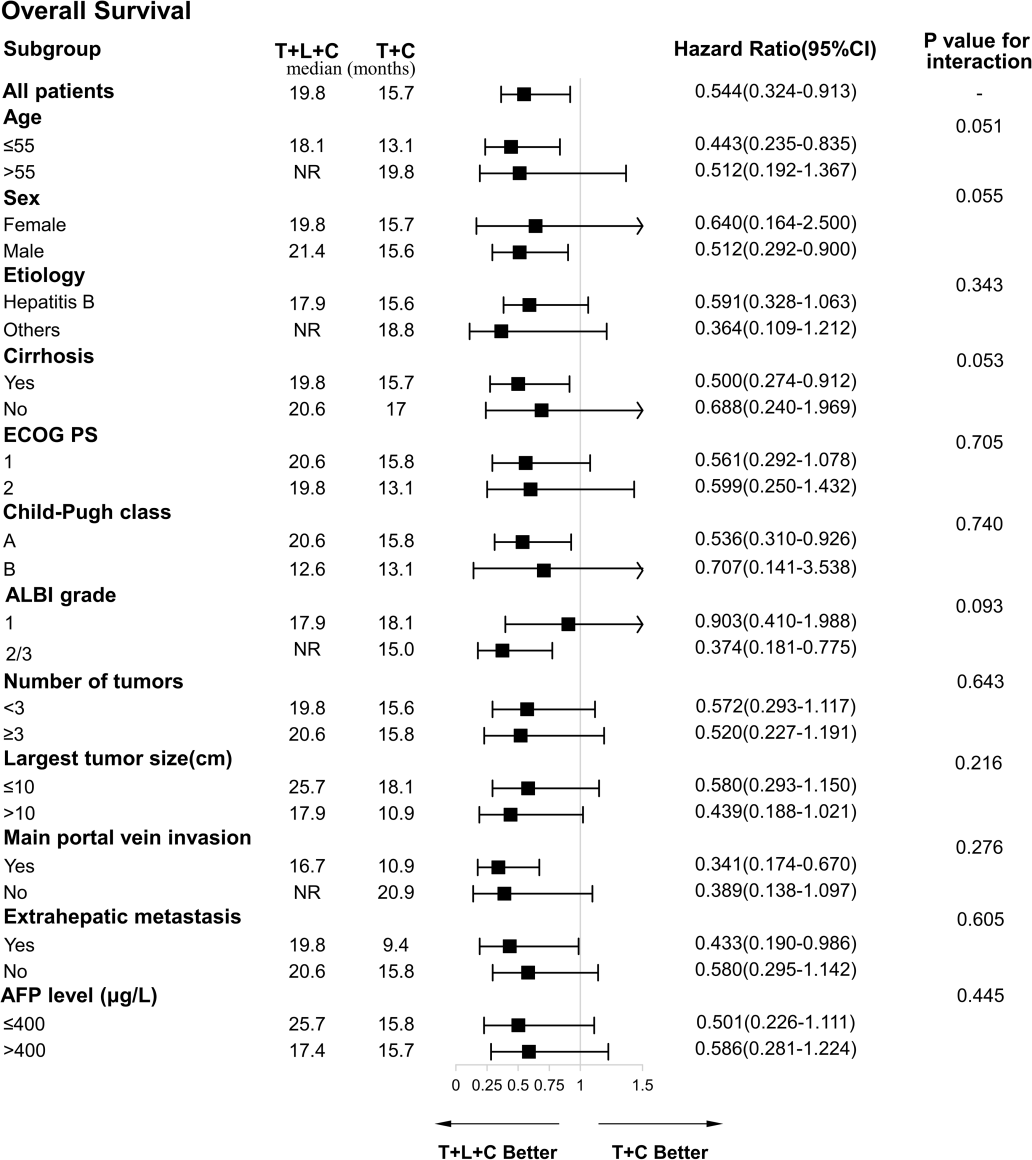
Forest plot of the subgroup analyses for overall survival. HR, hazard ratio; CI, confidence interval; ECOG PS, Eastern Cooperative Oncology Group Performance Status; ALBI, albumin-bilirubin; AFP, a-fetoprotein; T+L+C, transarterial chemoembolization combined with lenvatinib plus camrelizumab; T+C, transarterial chemoembolization combined with camrelizumab.

The ORR and DCR in the T + L + C group (ORR: 57.6%; DCR: 75.4%) were higher than in the T + C group (ORR: 41.5%; DCR: 58.5%) and the TACE group (ORR. 32.6%; DCR: 55.8%). There were no significant differences in the ORR and DCR between the T + L + C combination group and the T + C combination group (P > 0.05), whereas the differences in the ORR and DCR between the T + L + C combination group and the TACE group were statistically significant (P < 0.05) (**Table 2**). According to the results of the univariate and multivariate analyses (**Table 3**), the combination of T + L + C significantly improved PFS (HR,0.57; 95% CI,0.46–0.71; P < 0.001), in addition, patients with portal vein invasion had significantly shorter PFS (HR, 2.46; 95% CI,1.67–3.62; P < 0.001). Similarly, multivariate analysis of OS determined that T + L + C combination therapy significantly prolonged OS in patients (HR, 0.49; 95% CI, 0.39–0.62; P < 0.001), while the presence of portal vein invasion contributed negatively to OS in patients (HR, 2.54; 95% CI, 1.63–3.94; P < 0.001), whereas the treatment option and the presence of portal vein invasion were identified as independent prognostic factors for OS and PFS. The ECOG PS score, Child-Pugh classification, ALBI grade, extrahepatic metastasis, tumor number, largest tumor size, and AFP level were not significantly correlated with OS and PFS. Subgroup analysis of OS factors showed that compared to the T + C combination therapy group, OS did not differ significantly in outcomes across all subgroups, although T + L + C combination therapy achieved a longer OS in patients with portal vein invasion, extrahepatic metastasis, Child-Pugh classification A, ALBI grade 2/3, with cirrhosis, male sex, and age ≤55 years (**Figure 4**).

**Table 2:** Tumor response according to the mRECIST.

Characteristic	T+C+L vs T+C	P value	T+C+L vs TACE	P value
CR	9(15.8%) vs 3(7.3%)		9(15.8%) vs 1(2.3%)	
PR	24(42.1%) vs 14(34.1%)		24(42.1%) vs 13(30.2%)	
SD	10(17.5%) vs 7(17.1%)		10(17.5%) vs 10(23.3%)	
PD	14(24.6%) vs 17(41.5%)		14(24.6%) vs 19(44.2%)	
ORR	33(57.9%) vs 17(41.5%)	0.108	33(57.9%) vs 14(32.6%)	0.012
DCR	43(75.4%) vs 24(58.5%)	0.076	43(75.4%) vs 24(55.8%)	0.039

Data are presented as n (%). T+L+C, transarterial chemoembolization combined with lenvatinib plus camrelizumab; T+C, transarterial chemoembolization combined with camrelizumab; TACE, transarterial chemoembolization; mRECIST, modified Response Evaluation Criteria In Solid Tumors. CR, complete response; PR, partial response; SD, stable disease; PD, progressive disease; ORR, objective response rate; DCR, disease control rate.

**Table 3:** Analyses of prognostic factors for survival.

Factor	Overall survival				Progression-free survival			
	Univariate analysis		Multivariate analysis		Univariate analysis		Multivariate analysis	
	HR(95%CI)	P value	HR(95%CI)	P value	HR(95%CI)	P value	HR(95%CI)	P value
Treatment option								
T+L+C/T+C/TACE	0.47(0.38-0.60)	<0.001	**0.49(0.39-0.62)**	**<0.001**	0.57(0.46-0.71)	<0.001	**0.59(0.48-0.73)**	**<0.001**
Age	0.99(0.97-1.01)	0.297			1.00(0.98-1.02)	0.751		
Sex								
Female/Male	0.79(0.48-1.29)	0.346			0.66(0.42-1.03)	0.069	0.80(0.50-1.27)	0.342
Etiology								
Hepatitis B/Others	1.31(0.81-2.12)	0.273			1.21(0.78-1.87)	0.392		
Cirrhosis								
Yes/No	1.10(0.70-1.73)	0.687			1.22(0.80-1.86)	0.362		
ECOG PS score								
2/1	0.98(0.65-1.49)	0.929			1.08(0.73-1.58)	0.711		
Child-Pugh class								
B/A	1.26(0.65-2.44)	0.493			1.05(0.56-1.95)	0.889		
ALBI grade								
3/2/1	1.00(0.69-1.45)	0.983			0.97(0.69-1.37)	0.851		
portal vein invasion								
Yes/No	2.62(1.69-4.05)	<0.001	**2.54(1.63-3.94)**	**<0.001**	2.46(1.67-3.62)	<0.001	**2.46(1.67-3.62)**	**<0.001**
Extrahepatic metastasis								
Yes/No	1.07(0.71-1.62)	0.754			1.10(0.76-1.62)	0.610		
Number of tumors								
3/2/1	1.04(0.84-1.27)	0.749			0.98(0.81-1.18)	0.803		
Largest tumor size (cm)								
≥10/<10	1.00(1.00-1.01)	0.425			1.00(1.00-1.01)	0.499		
AFP level (μg/L)								
≥400/<400	1.25(0.84-1.85)	0.278			1.11(0.78-1.60)	0.563		

Analyses were performed using Cox proportional hazard regression model. HR, hazard ratio; CI, confidence interval; T+L+C, transarterial chemoembolization combined with lenvatinib plus camrelizumab; T+C, transarterial chemoembolization combined with camrelizumab; TACE, transarterial chemoembolization; ECOG-PS, Eastern Cooperative Oncology Group performance status; AFP, alpha-fetoprotein. HR and P values that are meaningful for multivariate analyses are in bold; bold is for eye-catching.

### Subsequent treatment after progression

Before the follow-up cut-off point, 35 cases in the combined T + L + C group showed disease progression, 26 patients required further treatment, and 7 patients chose supportive treatment; 34 cases in the combined T + C group showed disease progression, 25 patients required further treatment, and 7 patients selected supportive treatment; 38 cases in the TACE group showed disease progression, 26 patients took further treatment, and 6 patients chose supportive treatment. Due to limited treatment options after the progression of advanced HCC, some patients in three groups chose to abandon treatment (**Supplementary Table 2**).

### Safety results

The most common treatment-related AEs of any grade in patients in the T + L + C group were elevated total bilirubin (TB) (70.2%), fatigue (50.9%), hypertension (47.4%), weight loss (42.1%), thrombocytopenia (42.1%), and elevated aspartate aminotransferase (AST) (40.4%). Two patients in the T + L + C group had serious treatment-related emergency AEs, including one case of cerebral hemorrhage and one case of immune-related pneumonia, and no emergency adverse events (AEs) occurred in the other two groups (**Supplementary Table 1**). Dose reduction or interruption occurred in 16 (28.1%) and 11 (26.8%) patients in the T + L + C and T + C groups, respectively. We performed statistical analysis of biochemical indicators of liver function for the final recovery of the three groups of patients before the follow-up deadline, and only the laboratory indicator alanine aminotransferase (ALT) showed statistically significant differences between the groups (P = 0.014), while the remaining indicators AST, TB and ALB did not show statistically significant differences between the groups (P > 0.05), as shown in **Table 4**.

**Table 4:** Treatment-related adverse events in the three groups.

Adverse events	T+L+C(n=57)		T+C(n=41)		TACE(n=43)	
	All Grade	Grade3/4	All Grade	Grade3/4	All Grade	Grade3/4
Hypertension	27(47.4%)	1(1.8%)	6(14.6%)	0	0	0
fatigue	29(50.9%)	0	15(36.6%)	0	5(11.6%)	0
Nausea	7(12.3%)	0	5(12.2%)	0	5(11.6%)	0
Abdominal pain	13(22.8%)	0	8(19.5%)	0	2(4.7%)	0
Diarrhea	21(36.8%)	0	10(24.4%)	0	2(4.7%)	0
appetite decreased	18(31.6%)	0	12(29.3%)	0	8(18.6%)	0
Rash	16(28.1%)	1(1.8%)	11(26.8%)	0	0	0
Hand-foot syndrome	21(36.8%)	0	3(7.3%)	0	0	0
Mucositis	9(15.8%)	0	4(9.8%)	0	0	0
Hemorrhage	1(1.8%)	1(1.8%)	0	0	0	0
Arthritic pain	2(3.5%)	0	1(2.4%)	0	0	0
Weight loss	24(42.1%)	0	14(34.1%)	0	8(18.6%)	0
Leukopenia	16(28.1%)	1(1.8%)	6(14.6%)	0	6(14.0%)	0
Thrombocytopenia	24(42.1%)	5(8.8%)	11(26.5%)	2(4.9%)	17(39.5%)	0
Elevated TB	40(70.2%)	3(5.3%)	25(61.0%)	5(12.2%)	25(58.1%)	2(4.7%)
Elevated ALT	17(29.8%)	2(3.5%)	16(39.0%)	4(9.8%)	12(27.9%)	0
Elevated AST	23(40.4%)	7(12.3%)	24(58.5%)	4(9.8%)	20(46.5%)	2(4.7%)
Proteinuria	3(5.3%)	1(1.8%)	0	0	0	0
Hypothyroidism	5(8.8%)	0	1(2.4%)	0	0	0
Immune-related pneumonia	1(1.8%)	1(1.8%)	0	0	0	0

Data are presented as n (%). T+L+C, transarterial chemoembolization combined with lenvatinib plus camrelizumab; T+C, transarterial chemoembolization combined with camrelizumab; TACE, transarterial chemoembolization; TB, total bilirubin; ALT, alanine aminotransferase; AST, aspartate aminotransferase.

## Discussion

The results showed that patients treated with the T + L + C combination achieved longer OS and PFS compared to treatments with the T + C combination and TACE monotherapy. The T + L + C combination group also showed a better tumor response rate. We did not observe a significant difference in ORR and DCR between the T + L + C combination treatment and the T + C combination, but patients receiving the T + L + C combination achieved a better tumor response and longer survival, due to the long-term survival benefits provided by lenvatinib. We observed by further subgroup analysis that T + L + C combination therapy reduced the risk of death in the subgroup with portal vein invasion, extrahepatic metastasis, Child-Pugh classification A and cirrhosis.

The hepatic functional reserve is critical to allow patients to adhere to combination therapy to improve prognosis. Previous studies have reported that lenvatinib maintains hepatic functional reserve by slowing the progression of liver fibrosis (16), resulting in a survival benefit for patients with Child-Pugh classification A and cirrhosis. For patients with extrahepatic metastasis and portal vein invasion, our study demonstrated the need for lenvatinib in combination with TACE in addition to PD-1 inhibitors. Multifactorial analysis showed that both T + L + C combination therapy and patients without portal vein invasion were predicted to have better OS and PFS, whereas patients with HCC with portal vein invasion generally had a poor prognosis. One study (17) performed the TACE procedure combined with iodine-125 portal vein particle implantation to achieve a median survival time of 210 ± 17.5 days, whereas Ding et al. (18) combined TACE with lenvatinib to obtain an OS of 14.5 months. Although there are many approaches to the treatment of HCC with portal vein invasion, the best treatment modality has not yet been defined. However, encouragingly, our subgroup data showed that the mOS of patients with portal vein invasion treated with the T + L + C combination reached 16.7 months, which significantly improved the prognosis of these patients. Peng et al. (19) compared TACE in combination with lenvatinib with lenvatinib monotherapy in the treatment of patients with advanced HCC, resulting in a mOS of 17.8 months in the combination group and 11.5 months in the lenvatinib monotherapy group, showing a significant advantage of TACE in combination with lenvatinib in prolonging survival. The results of a meta-analysis involving 8,246 patients showed that TACE combined with tyrosine kinase inhibitors (TKIs) benefited HCC patients in terms of OS and tumor response rate compared to TACE (20).

In addition to combining TACE with lenvatinib, another approach that has been explored by a large number of investigators is the combination of TACE with ICIs. In recent years, some investigators have reported improved tumor response rates with camrelizumab combination for refractory TACE and confirmed a significant advantage of combination therapy over camrelizumab monotherapy (21, 22), which may be related to increased expression of PD-1 and PD-L1 in HCC after TACE, thus enhancing the antitumor activity of immune checkpoint inhibitors (23). The results of Brett Marinel et al. (12) also reported an encouraging set of survival data for multimodal immunotherapy with TACE, with a mOS of 35.1 months, a mPFS of 8.8 months, and downgraded disease in four patients, with access to liver transplantation. A multicenter randomized phase 2 trial of patients with advanced HCC treated with camrelizumab monotherapy for follow-up showed an ORR of 14.7% and a 6-month survival rate of 74.4%, demonstrating the antitumor activity and the initial survival benefit of camrelizumab (24). By exploring the two combination approaches, it was determined that both lenvatinib and camrelizumab contributed to the overall outcome of patients with HCC. Li et al. (25) compared lenvatinib in combination with camrelizumab with camrelizumab monotherapy, and the results showed that 1-year survival and PFS were significantly better in the combination group than in monotherapy group because lenvatinib improved the antitumor response to PD-1 inhibitors in addition to anti-angiogenesis. The underlying therapeutic rationale has not been fully elucidated, and it may be that lenvatinib combined with PD-1 inhibitors exert immunomodulatory effects by activating immune pathways, reducing regulatory T (Treg) cell infiltration, inhibiting transforming growth factor β (TGFß) signaling (26), and decreasing the proportion of monocytes and macrophages and increasing the proportion of CD8 + T cells to improve anti-PD-1 efficacy (27). In addition, lenvatinib reactivates interferon-gamma signaling in tumor cells by inhibiting fibroblast growth factor receptor (FGFR) (28) and enhances its combined antitumor activity with anti-PD-1 antibodies by blocking FGFR4 to reduce tumor PD-L1 levels and Treg differentiation (29).

The treatment pattern of TACE combined with lenvatinib and PD-1 inhibitors has been reported (30). A national multicenter retrospective study showed that TACE combined with PD-1 inhibitors and molecular targeted treatments (MTT) had significantly higher OS and PFS than TACE monotherapy (mOS, 19.2 vs. 15.7 months; P = 0.001; mPFS, 9.5 vs. 8.0 months; P = 0.002), with a mOS of 19 months and an ORR of approximately 60%, which were consistent the findings in our study (31). Furthermore, the results of a real-life clinical study showed that patients in the TACE combined with PD-1 inhibitors and the apatinib treatment achieved a mOS of 24.1 months and a mPFS of 13.5 months; this cohort had a better survival outcome than patients in our study, which we considered because the former study included 35.4% of HCC patients with BCLC stage B (32).

It should be noted that triple combination therapy significantly improves the survival outcome of patients with HCC who were in BCLC stage B or BCLC stage C. However, only a few studies have explored the application of triple combination therapy in patients with BCLC stage C HCC. Furthermore, our study was derived from real-life clinical practice. The results of a retrospective study of patients with hepatocellular carcinoma in BCLC stage C achieved mOS and mPFS of 16.9 months and 7.3 months, respectively, in the combined T + L + P group, and mOS and mPFS of 12.1 months and 4.0 month, respectively, in the combined T + L group (33). However, our results showed better survival outcomes than this cohort study, probably because our cohort included patients with a heavier tumor load, a higher proportion of patients with ≥3 tumors, and patients with tumor recurrence. In addition, the subgroup analysis of a previous cohort study showed that the T + L + P combination prolonged OS in patients without portal vein invasion, while our subgroup analysis showed the opposite results, suggesting that patients with portal vein invasion receiving the T + L + P combination had better OS than those receiving the T + P combination (16.7 months vs. 10.9 months, P = 0.001), whereas, in patients without portal vein invasion, there was no significant difference in OS between the two groups (P = 0.115). Due to the small sample size of these two retrospective studies, caution is warranted in interpreting the results of the subgroup analysis.

It is important to highlight the potential toxicity of combination regimens, including hepatic and renal toxicity, in addition to the significantly higher incidence of AEs such as hematological suppression. Additionally, more patients in the combined T + L + C group had hypertension (47.4%) and hand-foot syndrome (36.8%), which is consistent with that observed in previous studies. When counting the interval between TACE sessions, we found that the mean number of days between TACE sessions was lower in the TACE group than in the T + L + C combination group and the T + C combination group, suggesting that the combination therapy required less additional TACE therapy for the same survival period time and therefore the patients had sufficient time to recover liver function. In general, TACE combination drug therapy achieved good antitumor activity and a controlled safety profile.

The limitations of this study include that this was a retrospective study and patient treatment was determined by physician and patient selection, so we could not avoid effects due to selection bias and differences in clinical baseline characteristics on influencing treatment outcomes. Second, this study was conducted in a single institution with a small sample size of patients, and investigator bias and frequency of TACE procedures may have affected the results, which need to be further investigated in the future with large samples and prospective randomized controlled trials across multiple centers. In conclusion, our study showed that TACE combined with a PD-1 inhibitor and lenvatinib was more effective than TACE combined with a PD-1 inhibitor or TACE treatment alone in patients with stage C BCLC HCC.

## Data availability statement

The original contributions presented in the study are included in the article/**Supplementary Material**. Further inquiries can be directed to the corresponding author.

## Ethics statement

The requirement of ethical approval was waived by Ethics Committee, Tumor Hospital of Harbin Medical University for the studies involving humans because Ethics Committee, Tumor Hospital of Harbin Medical University. The studies were conducted in accordance with the local legislation and institutional requirements. The participants provided their written informed consent to participate in this study.

## Author contributions

JW, DH, and JZ were involved in the conception and design of the study. JW, HW, and XH provided the provision of study materials or patients. JW, HW, and ZH collected and assembled the data. JW, JZ, and DH were involved in data analysis and interpretation. JW, JZ, HW, and DH were involved in manuscript writing. All authors contributed to the article and approved the submitted version.
