# Supplementary material for: Efficacy and safety of transarterial chemoembolization combined with lenvatinib and camrelizumab in patients with BCLC-defined stage C hepatocellular carcinoma
Source: Front Oncol. 2023 Oct 17;13:1244341. doi: 10.3389/fonc.2023.1244341 (PMC10616839; doi:10.3389/fonc.2023.1244341)
Supplement: Supplementary file 1 [file DataSheet_1.pdf]

Supplementary Table 1 | TACE procedure-related parameters in the three groups

Parameters	T+L+C (n=57)	T+C (n=41)	TACE (n=43)	P value
**TACE technology**				0.481
cTACE	39(68.4%)	29(70.7%)	34(79.1%)	
DEB-TACE	18(31.6%)	12(29.3%)	9(20.9%)	
**Number of TACE**	4.0(3.0,5.0)	4.0(3.0,5.0)	3.0(2.0,4.0)	0.001
**Average days between TACE sessions**	78.3(55.5,124.1)	70.2(50.4,99.3)	62.0(45.0,81.7)	0.034
**Iodized oil dosage**	10.0(10.0,15.0)	10.0(5.0,10.0)	10.0(10.0,15.0)	
**Chemotherapy drugs**				
Pirarubicin	30.0(20.0,40.0)	30.0(20.0,35.0)	30.0(30.0,30.0)	0.879
Lobaplatin	50.0(0.0,50.0)	50.0(35.0,50.0)	50.0(50.0,50.0)	0.015
Raltitrexed	2.0(0.0,2.0)	2.0(0.0,2.0)	2.0(2.0,2.00)	0.190
**Laboratory metrics before treatment**				
AST	51.0(36.0,84.5)	44.0(32.0,64.0)	54.0(39.0,83.0)	0.219
ALT	37.0(26.0,55.5)	34.0(24.0,50.0)	37.0(26.0,52.0)	0.677
TB	20.8(15.2,24.6)	18.6(13.0,32.1)	20.1(16.9,28.1)	0.693
ALB	38.98±5.11	39.39±4.70	39.38±3.93	0.880
**Laboratory metrics after treatment**				
AST	56.0(45.0,140.0)	72.0(45.0,120.5)	58.0(39.9,97.0)	0.276
ALT	46.0(30.0,103.0)	40.0(27.0,94.5)	30.0(23.0,44.0)	0.014
TB	27.2(19.9,34.8)	25.5(18.4,50.5)	26.7(17.9,37.4)	0.719
ALB	35.98±5.05	36.81±4.57	36.68±4.30	0.634

Data are presented as mean ± standard deviation or n (%) or median (25th-75th), cTACE, conventional transarterial chemoembolization; DEB-TACE, drug-eluting beads transarterial chemoembolization; AST, aspartate aminotransferase; ALT, alanine aminotransferase; TB, total bilirubin; ALB, albumin. T+L+C, transarterial chemoembolization combined with lenvatinib plus camrelizumab; T+C, transarterial chemoembolization combined with camrelizumab; TACE, transarterial chemoembolization.

Supplementary Table 2 | Subsequent treatment after progression.

	T+L+C (n=35)	T+C (n=34)	TACE (n=38)
TACE+Lenvatinib+Camrelizumab	3	0	0
Lenvatinib+ Camrelizumab	5	3	3
Lenvatinib	4	6	7
Apatinib	4	1	0
Donafenib	2	1	0
Regorafenib	4	0	0
Radiotherapy	1	0	0
HAIC	0	5	0
TACE+ Camrelizumab	0	3	0
Camrelizumab r	0	0	2
TACE+Lenvatinib	0	0	2
TACE+ Sorafenib	0	0	1
Sorafenib	0	0	7
Sorafenib+ Camrelizumab	0	0	2
TACE	3	6	3
Supportive Care	7	7	6
Abandonment of treatment	2	2	5

TACE, transarterial chemoembolization; HAIC, hepatic arterial infusion of chemotherapy.
